# Discovery and functional characterization of endoglucanases from *Coptotermes formosanus* with enhanced cellulose hydrolysis via yeast surface display

**DOI:** 10.1128/aem.00682-25

**Published:** 2026-03-02

**Authors:** Jingfang Sun, Chang Cao, Yushi Chen, Tian Shi, Shuai Guo, Lingling Zhu, Shaodong Wang, Jinlei Bian, Lin Yang

**Affiliations:** 1 School of Basic Medicine and Clinical Pharmacy, China Pharmaceutical University 540420 , Nanjing, Jiangsu, China; 2 The Nanjing Termite Control Institute, Nanjing, Jiangsu, China; 3 Affiliated Nanjing Jinling Hospital, School of Medicine, Nanjing University 12581, Nanjing, Jiangsu, China; 4 School of Pharmacy, China Pharmaceutical University 428683 , Nanjing, Jiangsu, China; Washington University in St. Louis, St. Louis, Missouri, USA

**Keywords:** cellulose hydrolysis, *Saccharomyces cerevisiae*, yeast surface display, *Coptotermes formosanus*, endoglucanases

## Abstract

Microbial cellulose degradation offers a sustainable route to convert agricultural and forestry residues into value-added chemicals. Here, we report the discovery and functional characterization of eight novel endoglucanases (CTEG1 and CTEG3–CTEG9) from a *Coptotermes formosanus* cDNA library and heterologously expressed in *Saccharomyces cerevisiae*. Screening of crude extracts and whole-cell fermentations identified CTEG6 as the top performer (≈30 U/mg at 30°C) and the best whole-cell hydrolytic strain (1.51 mg/mL reducing sugar after 72 h). To investigate the basis of activity variation, we quantified EG gene copy number by quantitative PCR (qPCR) and observed strain-to-strain differences in gene dosage. Structural modeling (AlphaFold2) and flexible induced-fit docking with a carboxymethyl cellulose sodium (CMC-Na) fragment implicated residues A11, D251, and S258 in substrate binding; targeted mutagenesis of these sites (CTEG10: A11S/D251N/S258G) reduced hydrogen-bonding *in silico* and substantially lowered hydrolytic activity both *in vitro* and in 72-h fermentations. Under optimized conditions (30°C, pH 6.0), the CTEG6 strain efficiently hydrolyzed CMC-Na, microcrystalline cellulose (MCC), and natural cellulose from corncob powder. Together, these results identify CTEG6 as a promising biocatalyst and provide a theoretical basis for rational enzyme optimization toward scalable biomass conversion.

**IMPORTANCE:**

Efficient, low-impact methods to depolymerize cellulose are essential for turning agricultural and forestry residues into renewable chemicals and materials. Here, we report eight novel endoglucanases mined from a termite cDNA library and show that yeast surface display both enhances cellulose hydrolysis and enables enzyme reuse, identifying CTEG6 as a particularly robust candidate for further development. By integrating enzyme characterization, molecular docking, targeted mutagenesis, and fermentation optimization, we pinpoint specific residues (A11, D251, and S258) as key contributors to substrate binding and catalytic performance. These mechanistic insights not only justify prioritizing CTEG6 for further applications but also offer a practical route toward more efficient, sustainable bioconversion technologies for renewable energy, waste valorization, and green manufacturing.

## INTRODUCTION

With the depletion of fossil resources and the escalation of environmental challenges, it has become increasingly urgent to identify renewable feedstocks for the synthesis of high-performance, eco-friendly polymers (1). Biomass polymers, products of plant photosynthesis, constitute the Earth’s most abundant renewable resource, with an estimated annual yield of 1.5 × 10¹² tons (2, 3). Cellulose, the principal component of plant cell walls, represents a major fraction of this biomass and ranks among the most plentiful biopolymers available (2, 4). Comprised of over 10,000 anhydro-glucose units (5), cellulose can be enzymatically hydrolyzed to glucose, which in turn serves as a versatile platform for producing valuable chemicals via hydrolysis, hydrogenolysis, hydrogenation, dehydration, oxidation, isomerization, or retro-aldol condensation pathways (6–8). However, the rigid, tightly bound structure of lignocellulosic biomass in straw and wood renders chemical pretreatment both energy-intensive and expensive (9). In contrast, microbial degradation offers a highly efficient, low-cost, and environmentally benign alternative, presenting significant promise for sustainable bioconversion processes (10, 11).

Cellulolytic activity relies on a synergistic, multicomponent enzyme system, endoglucanase (EG), exoglucanase (cellobiohydrolase), and β-glucosidase, rather than on a single monomeric enzyme (12, 13). Among these, EG plays a pivotal role by targeting the amorphous regions of cellulose fibers, randomly cleaving β-1,4-glycosidic bonds to shorten polymer chains, generate new chain ends, and release soluble oligosaccharides (14–16). Thus, identifying endoglucanases with high catalytic efficiency and broad substrate adaptability is essential for effective biodegradation of native cellulose.

Termites are essential decomposers of cellulosic material in nature (17). Their cellulose digestion relies on two complementary systems: endogenous cellulases produced by the termite itself and cellulases from symbiotic bacteria and protists in the gut (18–20). The combination of termite morphology, gut structure, and diverse microbial communities enables efficient breakdown of lignocellulose (21). Numerous studies have demonstrated the high enzymatic activity and broad adaptability of termite-derived endoglucanases (22, 23). In particular, *Coptotermes* (*C*.) *formosanus*, a widespread species renowned for its potent cellulose-degrading ability, has been the focus of genetic investigations into cellulose-degradation enzymes (24, 25).

*Saccharomyces* (*S*.) *cerevisiae* is a promising host for cellulose degradation engineering, given its remarkable tolerance to the toxic compounds inherent in natural biomass and its suitability for large-scale industrial processes (26, 27). However, native cellulose polymers are too large to traverse the yeast cell wall unaided (27). To overcome this obstacle and lower energy inputs, yeast surface display technology has been developed to anchor and present heterologous enzymes on the cell wall (28). *S. cerevisiae* surface-display systems typically use either agglutinin or flocculin proteins as the fusion partners to anchor heterologous enzymes to the cell wall (29). Flocculin *FLO8* contains an N-terminal flocculation domain that binds cell-wall glycoproteins and a C-terminal GPI anchor region that tethers it to the membrane and cell wall (30, 31). By fusing target enzymes to Flo8, researchers have stably displayed various cellulolytic proteins on the yeast surface using the flocculin system (32, 33).

EG is a key enzyme in converting cellulose into urgently needed energy, food, and chemical raw materials in an environmentally friendly and efficient manner (34). Therefore, isolating highly active, thermostable, and broadly adaptable EGs from *C. formosanus* cDNA libraries, coupled with flocculin surface display technology and systematic optimization of reaction conditions, is crucial for harnessing natural cellulose to alleviate resource constraints.

## MATERIALS AND METHODS

### Biological samples and gene cloning

Samples of *C. formosanus* were collected in Chaohu City, Anhui Province, on 30 March 2023, by the Nanjing Termite Control Research Institute and subsequently provided by Tian Shi (Nanjing, China). Termites were maintained on Masson’s pine blocks for at least 1 week before experiments. Total RNA was extracted using the TRIzol reagent, and first-strand cDNA was synthesized with a Takara reverse transcription kit (Takara, Shiga, Japan) (35). Based on conserved domains and homologous sequences of known *C. formosanus* endoglucanase genes, gene-specific primers incorporating appropriate restriction sites were designed (36). To construct the yeast display vector, the MF.SS signal peptide and the C-terminal GPI-anchoring domain of Flo8 were amplified from *S. cerevisiae* BY4741 cDNA (prepared in our laboratory) using primers targeted to the BY4741 genome (30, 37). All primers used for plasmid construction are listed in Table 1.

**TABLE 1:** Gene-specific primers used in this study

Primers	Sequence (5′–3′)
CTEGn-F (KpnI)	CGGGGTACCGCTTACGACTACAAGACAGTACTGAAGA
CTEGn-R (SacII)	TCCCCGCGGTTACACGCCTGCCTTGAGGAGACC
MF.SS-F (BamHI)	CGCGGATCCATGAGATTTCCTTCAATTTTTACTGCAG
MF.SS-R (KpnI)	CAAACGAAGACCCTGGTACCTCTTTTATCCAAAGATACCCCT
FLO8-F (SacII)	TCCCCGCGGTCGTATCCAGATTCAATTCCTCCC
FLO8-R (NheI)	CTAGCTAGCGAGATCGTAATCCGGTCCTTG
Actin-F	CTTCGAACAAGAAATGCAAACCG
Actin-R	GGCAGATTCCAAACCCAAAACAG
EG-qPCRF	CATGGGTAAGGAAAAGACTCACG
EG-qPCRR	GCACCTGATTGCCCGACATTATC
CTEG10-F1	TGATTTCCGAAGAAGACCTCGAGTAAGCTTGCTTACGACTACAAGACAGTACTGAAGAATTCTCTGCTTTTCTACGAGGC
CTEG10-R1	TTGGCCAGTAGAACCTGTACTCCGGAGATCTTGTTATCCCAGTTGAAGGCACCATTCCAG
CTEG10-F2	GAAGCTGGAATGGTGCCTTCAACTGGGATAACAAGATCTCCGGAGTACAGGTTCTACTGG
CTEG10-R2	TCGGTTAGAGCGGATCTTAGCTAGCCGCGGTTACACGCCTGCCTTGAGGAGACCGGCGAC

### Plasmid construction, site-directed mutagenesis, and yeast transformation

The plasmid pCEV-GM1 was optimized in our lab (China Pharmaceutical University Biomedicine R&D Transformation Platform) from the original pCEV plasmid, containing a strong *PGK1* promoter and a green fluorescent protein (GFP) reporter for monitoring surface display effects (38). Using restriction digestion and ligation, gene cassettes were assembled as follows: the *MF.SS* fragment (BamHI/KpnI) was inserted downstream of *PGK1*; the *EG* fragment (KpnI/SacII) was cloned downstream of *MF.SS*; and the *FLO8* fragment (SacII/NheI) was positioned between the EG insert and the *CYC1* terminator. This workflow generated two expression formats for each EG variant: *PGK1–MF.SS–CTEGn–FLO8* for surface display and *PGK1–MF.SS–CTEGn* for soluble expression controls.

Targeted point mutations (A11S, D251N, and S258G) were introduced into the CTEG6 coding sequence by nested PCR to produce the triple-mutant cassette *CTEG6^A11S,D251N,S258G^* (hereafter CTEG10). Briefly, overlapping fragments carrying the intended substitutions were amplified and fused by a nested PCR to generate the full-length mutant insert, which was cloned into the pCEV-GM1 backbone following the same cloning scheme described above. Mutagenic primer sequences are listed in Table 1.

The *S. cerevisiae* strain CEN.PK2-1C, selected for its robust growth and genetic tractability, was obtained from a commercial supplier (39, 40). Recombinant plasmids (wild type and mutant) were transformed into *S. cerevisiae* cells via the LiAc/SS-DNA/PEG method (40, 41). Transformants were screened by colony PCR and verified by Sanger sequencing (GenScript Biotech, Nanjing, China). Sequence alignments were performed by the MEGA software. Confirmed strains harboring each EG construct were inoculated into YPD medium (10 g/L yeast extract, 20 g/L peptone, 20 g/L glucose as a carbon source) at 30°C for subsequent expression, activity assays, and fermentation experiments.

### Laser confocal microscopy detection

Laser confocal microscopy was used to verify the actual effect of surface display expression cassette. Yeast cultures were grown for 72 h, then cells were harvested by centrifugation at 3,000 rpm and washed three times with PBS. Pellets were resuspended in PBS, and 20 μL aliquots were mounted on slides. Images were acquired via an FV1000 laser scanning confocal microscope (Olympus, Tokyo, Japan).

### Crude enzyme extraction and activity assay

Yeast cultures were grown in YPD medium at 30°C until an OD_600_ of 10 was reached. One milliliter aliquot of the cell suspension was pelleted by centrifugation at 3,000 rpm for 5 min, and the supernatant was discarded. The cells were washed 2–3 times with 1× PBS to remove residual medium and debris. The washed cells were then disrupted in a high-throughput tissue grinder, and the lysate was clarified by filtration through a sterile 0.45 µm membrane to yield the crude enzyme extract. For activity measurements, 2% (wt/vol) carboxymethyl cellulose sodium (CMC-Na) was prepared in 20 mM sodium phosphate buffer (pH 6.0) and incubated with the crude enzyme extract at 30°C for 1 h. To assess thermal stability, parallel reactions were conducted at 50°C. Reducing sugars released were quantified by the DNS method (42), with absorbance measured at 540 nm on a Tecan M2000pro microplate reader (Tecan, Männedorf, Switzerland). Specific activity of EG is expressed as enzyme activity per milligram of yeast cell dry weight. One unit of cellulase activity is defined as the amount of enzyme that releases 1 µmol of reducing sugar from CMC-Na per minute under the assay conditions.

### Viable cell-based cellulose degradation assay

Engineered yeast strains were precultured in YPD medium for 12 h at 30°C to an OD_600_ of 0.6–0.8. Cells were then harvested and used to inoculate batch fermentations in specialized medium (10 g/L yeast extract, 20 g/L peptone, and 20 g/L CMC-Na carbon source). Fermentation cultures were incubated at 30°C with shaking at 220 rpm and sampled at 12, 24, 36, 48, 60, 72, and 96 h. Prior to analysis, the sample pH was adjusted to 6.0. To identify the optimal conditions for cellulose degradation, we systematically varied fermentation temperature, initial medium pH, and substrate type, comparing the performance of strains expressing different *EG* genes.

### Saccharification of natural corncob cellulose

Corncob powder, sieved through a 40-mesh screen, was used to formulate the natural cellulose degradation medium (10 g/L yeast extract, 20 g/L peptone, 20 g/L corncob powder as carbon source). Engineered strains were inoculated into the medium and incubated at 30°C for 96 h, with samples taken at fixed intervals and centrifuged. The supernatant was used to measure released reducing sugars via the DNS method. Reducing sugar concentration was reported as milligrams of sugar released per milliliter of reaction mixture.

### Quantitative PCR-based determination of EG gene copy number

Genomic and plasmid DNA were extracted from engineered *S. cerevisiae* cultures using a commercial yeast genomic DNA isolation kit and a plasmid extraction kit, respectively (Yeasen Biotechnology, Shanghai, China). Quantitative PCR (qPCR) was performed with a SYBR Green-based master mix on ViiA7 (Thermo Fisher, Waltham, MA). Reactions (20 µL) contained 1xSYBR Green master mix, 0.2 µM of each primer (Table 1), and 2 µL template DNA. Thermal cycling consisted of an initial denaturation at 95°C for 3 min, followed by 40 cycles of 95°C for 10 s and 60°C for 30 s, and a final melt-curve analysis to confirm product specificity. Relative copy number of the EG was calculated using the comparative Ct method: = 2^(Ct^_^EG^_^−Ct^_^Actin^_^)^, where Ct_Actin_ is the cycle threshold for the single-copy Actin, and Ct_EG_ is the Ct for the EG amplicon. Reported copy numbers are the mean ± SD of three biological replicates and are rounded to the nearest integer for presentation.

### Molecular docking and protein structure analysis

To investigate structural determinants underlying activity differences among EGs, three-dimensional models of CTEGn were predicted using AlphaFold2 via the ColabFold server (MMseqs2 mode, v1.3.0) (43). Structural differences were inspected and compared using the SWISS-MODEL website (44). For ligand docking, representative CMC-Na fragments were retrieved from PubChem. Docking runs were performed with AutoDock v4.2.6, then top-scoring poses were selected for further inspection. Docked complexes were visualized and analyzed in ChimeraX and BIOVIA Discovery Studio to map hydrogen bonds, hydrophobic contacts, and substrate-binding clefts (45). The optimal scoring results were utilized to display the initial geometric shapes of the molecular mechanics optimization for the complexes formed by the binding of CTEGn with CMC-Na, aiming to investigate the relative binding strengths and substrate-accommodation features across EG variants.

## RESULTS

### Identification and sequencing of *CTEGn* endoglucanase genes

**Fig 1:**
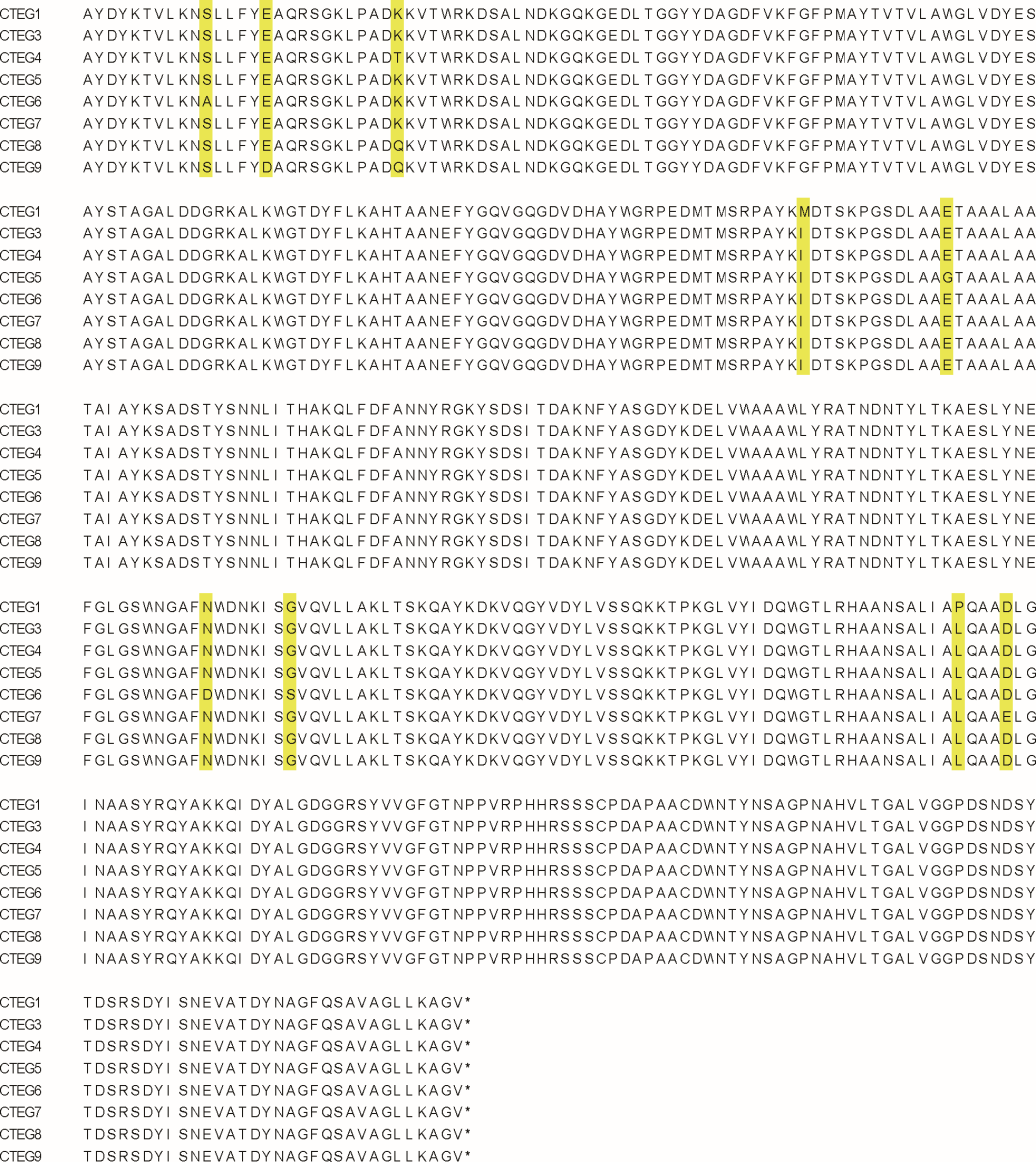
Alignment of the amino acid sequences of the eight cellulases CTEGn (*n* = 1 and 3–9), with variable sites highlighted in yellow.

Total RNA of *C. formosanus* samples was extracted and reverse-transcribed to cDNA, from which eight distinct *EG* gene sequences were identified, designated as *CTEGn* (*n* = 1 and 3–9) (Fig. 1). Each *EG* gene contains a 1,299 bp open reading frame encoding a 432-amino-acid protein with a predicted molecular weight of ~47 kDa.

### Construction and functional validation of a Flo8-based yeast surface display for CTEG1

**Fig 2:**
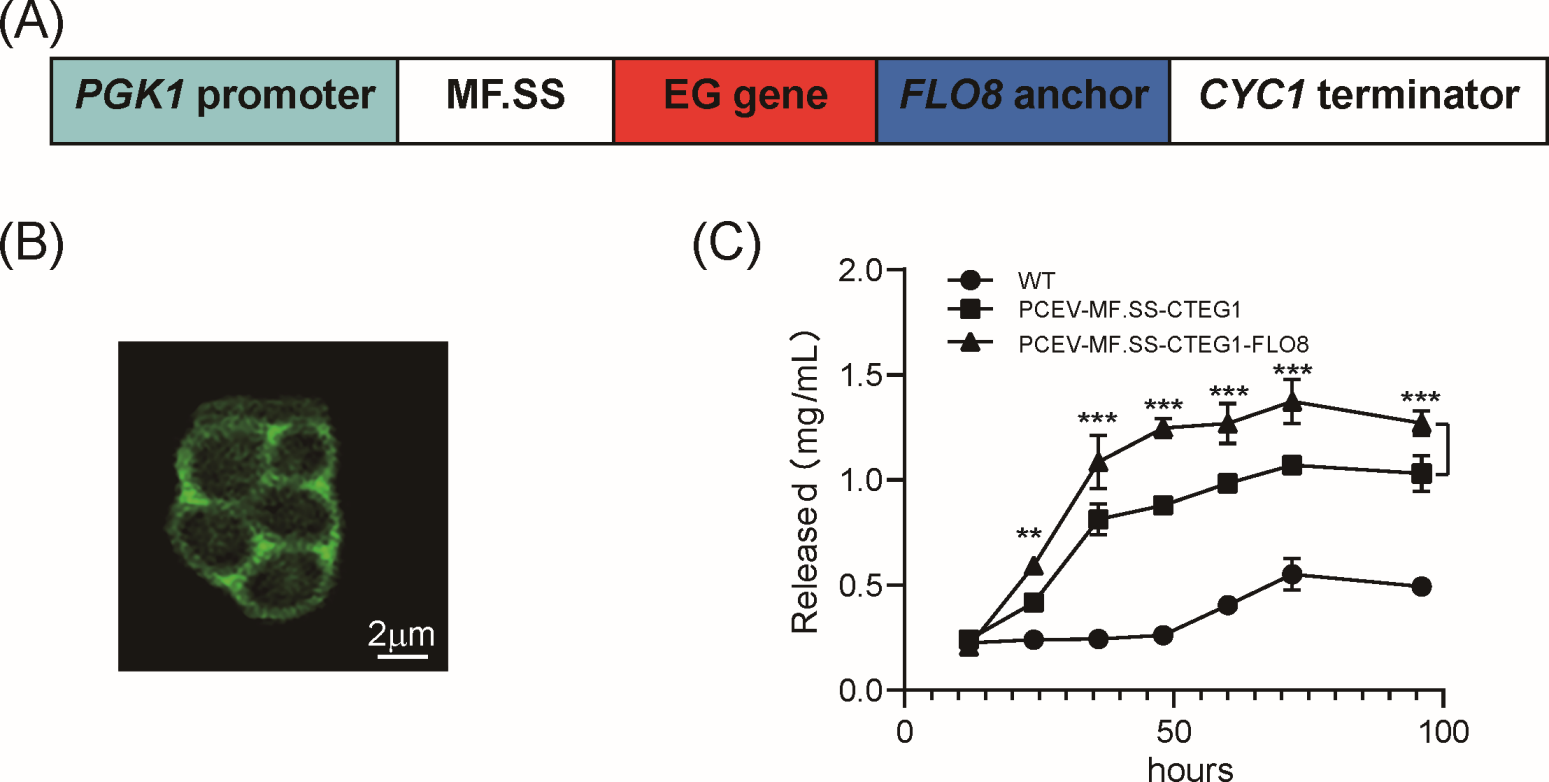
Surface-display design and evaluation. (**A**) Schematic of the gene cassette used for surface display, comprising the secretion signal peptide *MF.SS*, the *EG* insert, and the *FLO8* flocculin anchor. (**B**) Detection image using laser confocal microscopy, visually demonstrating the effect of surface display through the distribution of GFP (representative image; scale bar = 2 µm). (**C**) Time course of reducing-sugars release from 20 g/L CMC-Na during fermentation of three strains: wild type, CTEG1 expressed without anchor, and CTEG1 displayed on the cell surface. Data are shown as mean ± SD (*n* = 3). At each sampled time point, enzyme activity in the surface-display group (*PCEV–MF.SS–CTEG1–FLO8*) was compared to the free-expression control (*PCEV–MF.SS–CTEG1*) using two-tailed unpaired Student’s *t*-tests. Differences were considered statistically significant at *P < 0.05; **P  < 0.01; ***P  < 0.001.

The haploid yeast strain *S. cerevisiae* CEN.PK2-1C was transformed with our engineered pCEV-GM1 plasmid to assemble a Flo8-based cell-surface display cassette consisting of the strong *PGK1* promoter, the *MF.SS* secretion signal peptide, and the *FLO8* flocculin anchor (Fig. 2A) (46). To validate its functionality, we displayed the novel endoglucanase CTEG1 as a test case. Laser confocal microscopy revealed that the GFP reporter localized predominantly to the cell wall, confirming successful anchoring of the EG enzyme (Fig. 2B). EGs act preferentially on the amorphous regions of cellulose, randomly hydrolyzing β-1,4-glucosidic bonds and ultimately producing soluble oligosaccharides (primarily reducing sugars).

Next, we compared the cellulose-hydrolysis performance of the surface-display strain (pCEV*–MF.SS–CTEG1–FLO8*) versus a free-enzyme control (pCEV*–MF.SS–CTEG1*) by quantifying released reducing sugars. Although both strains began with similar sugar titers, the surface-display strain achieved a significantly higher reducing-sugar concentration at 72 h, 1.3747 mg/mL compared to 1.0714 mg/mL in the control (Fig. 2C). After peaking at 72 h, reducing-sugar levels declined, likely reflecting increased cellular uptake and utilization of released oligosaccharides. Collectively, these results demonstrate that anchoring CTEG1 to the yeast surface markedly enhances cellulose-degradation efficiency, validating the effectiveness of the Flo8-based surface-display system.

### *In vitro* and *in vivo* evaluation of endoglucanase performance

**Fig 3:**
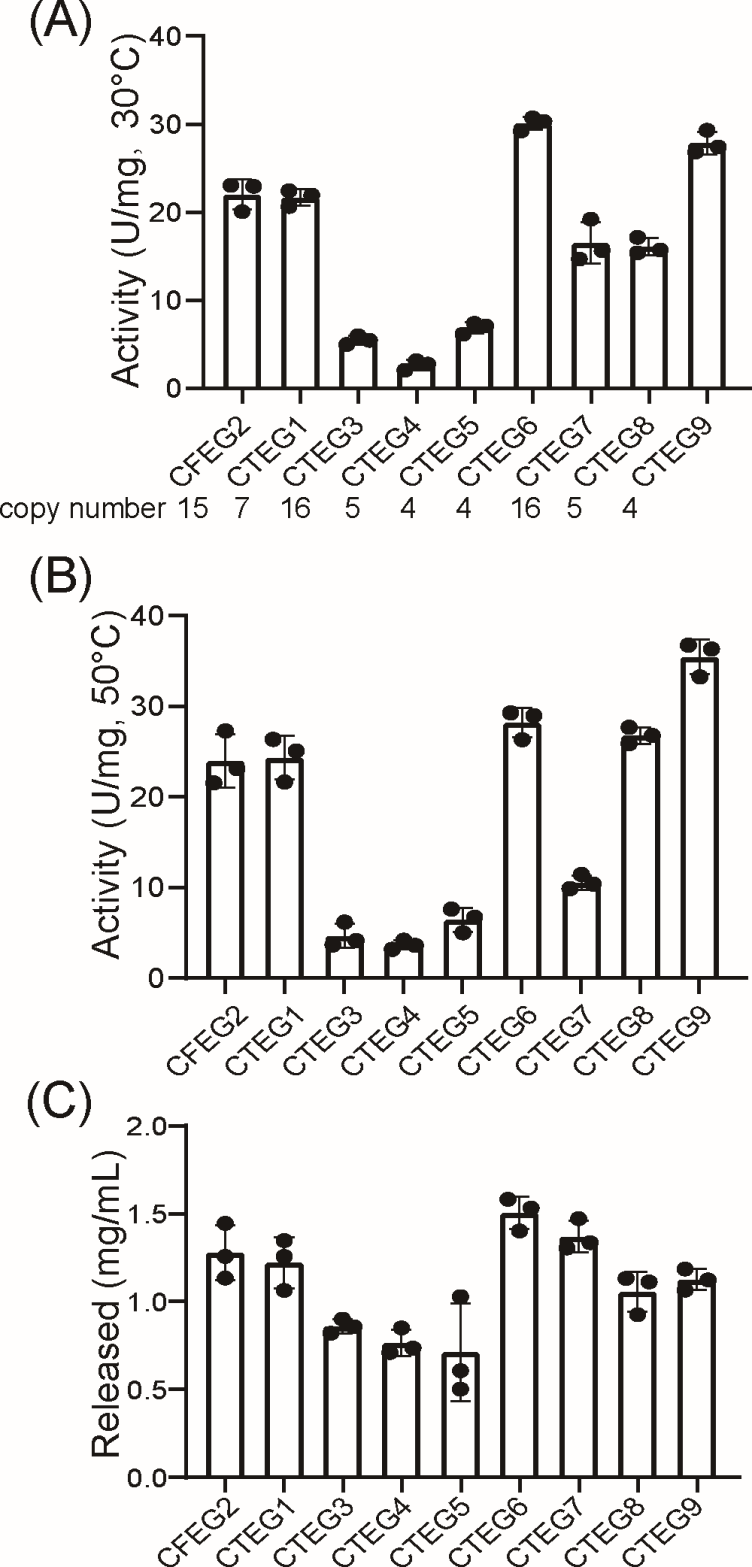
Detection of enzyme activity and shake flask fermentation of endoglucanases. Enzyme activities of nine EGs (CFEG2, CTEG1, CTEG3–CTEG9) were measured by crude enzyme solutions on 20 g/L CMC-Na at 30°C (**A**) and 50°C (**B**). A previously reported endoglucanase (CFEG2) was included as a positive control. Activities were determined by the DNS assay and are expressed as U/mg (dry cell weight). *EG* gene copy numbers for each strain are indicated beneath the x-axis in panel A. (**C**) The titer of reducing sugar after 72 h of fermentation. Data are shown as mean ± SD (*n* = 3).

Crude enzyme extracts from each engineered strain, harvested at an OD_600_ of 10, were assayed for endoglucanase activity by quantifying the conversion of substrate CMC-Na into reducing sugars. Under the reaction conditions at 30°C, CTEG6 exhibited the highest enzymatic activity (30.09 U/mg), followed by CTEG9 (27.86 U/mg) and CTEG1 (21.69 U/mg) (Fig. 3A). However, when the reaction temperature was raised to 50°C, CTEG9’s activity increased markedly to 35.44 U/mg, surpassing all other variants. CTEG6 (28.20 U/mg) and CTEG1 (24.35 U/mg) also retained high activity at the elevated temperature, whereas the remaining EGs displayed variable temperature tolerance and activity profiles (Fig. 3B). A previously reported endoglucanase (CFEG2) was included as a positive control (47). These results demonstrate that different endoglucanase homologs possess distinct optimal temperatures and thermostability characteristics.

Subsequently, live cell fermentation was performed using each of the eight EGs strains with 2% CMC-Na as the carbon source. After 72 h of fermentation, the reducing-sugar titers mirrored the *in vitro* activity profiles, with the CTEG6 strain again exhibiting the highest hydrolytic performance at 1.51 mg/mL (Fig. 3C). To assess whether gene dosage contributed to the activity differences observed among transformants, we quantified EG copy number in all nine (CFEG2, CTEG1, CTEG3–CTEG9) engineered *S. cerevisiae* strains by qPCR using *Actin* as a single-copy reference. These data indicate that the measured enzymatic activities reflect a combination of intrinsic catalytic properties and gene-dosage effects; accordingly, we note copy number as a potential contributor when interpreting activity differences (Fig. 3A). In future work, we will minimize this variable by integrating single-copy constructs at defined genomic loci using targeted genome-editing approaches. Based on these comprehensive data, the *S. cerevisiae* strain expressing *CTEG6* was chosen as the most effective endoglucanase for all subsequent experiments.

### Optimization of cellulose fermentation conditions and degradation of natural cellulose

**Fig 4:**
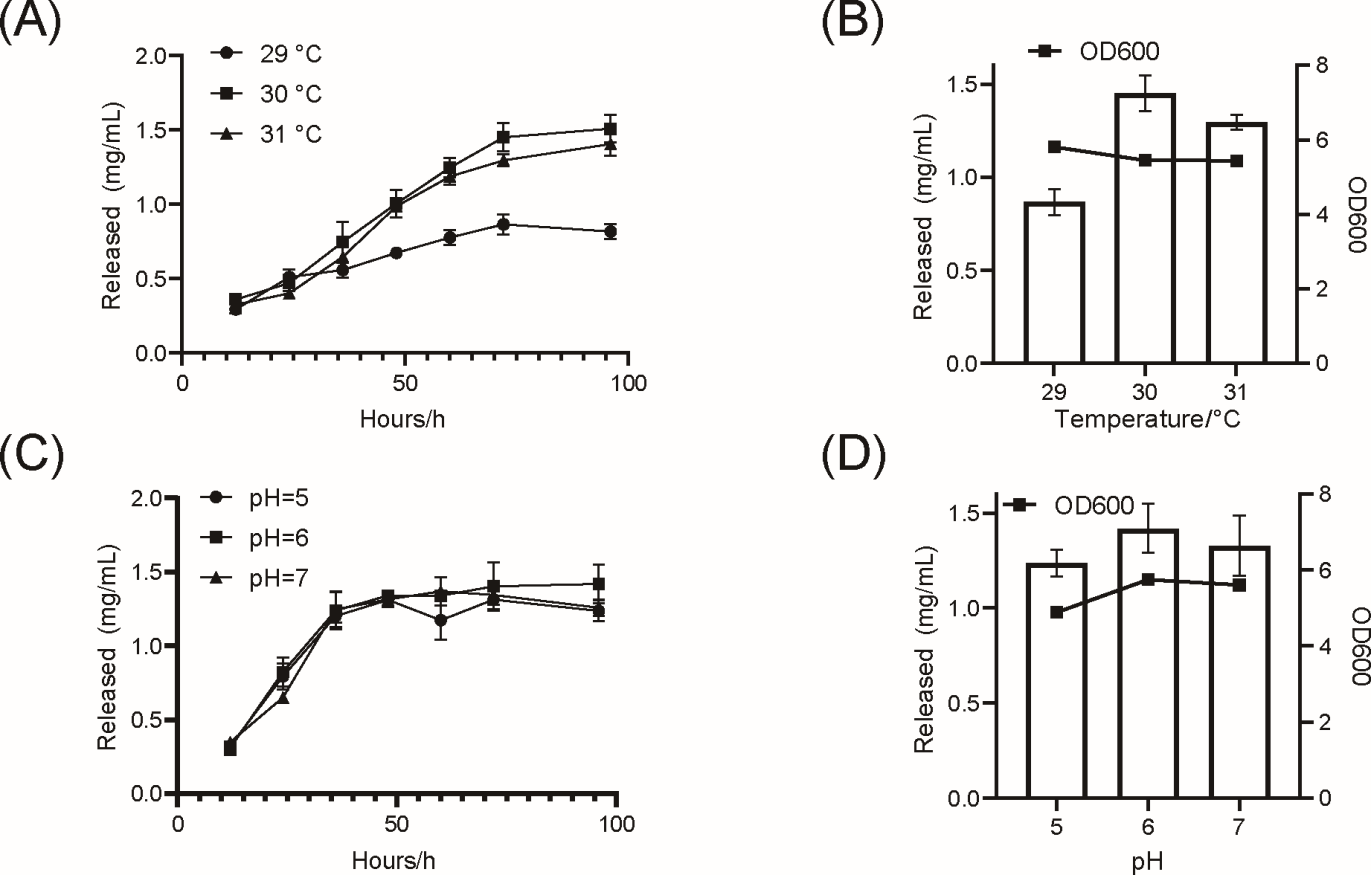
Optimal fermentation temperature and pH for the CTEG6 surface-displaying strain. (**A**) Time course of reducing sugar release from 20 g/L CMC-Na during fermentation at different temperatures, illustrating temperature-dependent effects on cellulose hydrolysis. (**B**) Comparison of reduced-sugar titer and culture density (OD_600_) at 72 h across the tested temperatures. (**C**) Time course of reducing sugar released from 20 g/L CMC-Na during fermentation as the substrate at different pH values. (**D**) Comparison of reducing-sugar titer and culture density (OD_600_) at 72 h across the tested pH conditions, with pH 6.0 yielding the highest values under our experimental conditions. Data are presented as mean ± SD (*n* = 3). Reducing sugars were measured by the DNS assay and are expressed as mg/mL.

To optimize cellulase production, fermentation conditions for the engineered *S. cerevisiae* strain expressing the *CTEG6* gene were systematically evaluated using CMC-Na (20 g/L) as the substrate. Temperature optimization revealed that 30°C was the most favorable condition, yielding the highest cellulase activity. Lower temperatures markedly reduced enzymatic performance, while higher temperatures inhibited yeast growth and impaired cellulose degradation (Fig. 4A). After 72 h, cell density (OD_600_) and reducing-sugar titers at 29°C, 30°C, and 31°C were compared (Fig. 4B). At 29°C, OD_600_ reached 5.81 with 0.86 ± 0.05 mg/mL reducing sugar; at 30°C, OD_600_ was 5.45 with 1.45 ± 0.08 mg/mL; and at 31°C, OD_600_ remained at 5.44 but reducing sugar fell to 1.29 ± 0.03 mg/mL (Fig. 4B). Although cell growth was slightly higher at 29°C, hydrolytic capacity declined, indicating that cooler temperatures impair CTEG6 activity, whereas warmer conditions stress yeast viability. Thus, 30°C offers the optimal compromise between biomass accumulation and cellulose degradation for this engineered strain.

Under the optimal temperature of 30°C, the strain exhibited similarly high reducing-sugar titers across pH 5–7 during the first 60 h (Fig. 4C). After 60 h, however, the rate of sugar depletion diverged by pH, indicating pH-dependent effects on hydrolysis efficiency. After 72 h, the culture at pH 5.0 achieved an OD_600_ of 4.89 and released 1.31 ± 0.08 mg/mL reducing sugar; at pH 6.0, OD_600_ rose to 5.75 with 1.41 ± 0.19 mg/mL sugar; and at pH 7.0, OD_600_ was 5.61 with 1.37 ± 0.10 mg/mL (Fig. 4D). These results demonstrate that pH 6.0 offers the best compromise between cell growth and sugar production. These observations align with previously reported *in vitro* activity profiles of EGs (48), confirming that temperature and pH are key determinants of fermentation performance. Accordingly, all subsequent cellulose-degradation experiments were carried out under 30°C and pH 6.0.

**Fig 5:**
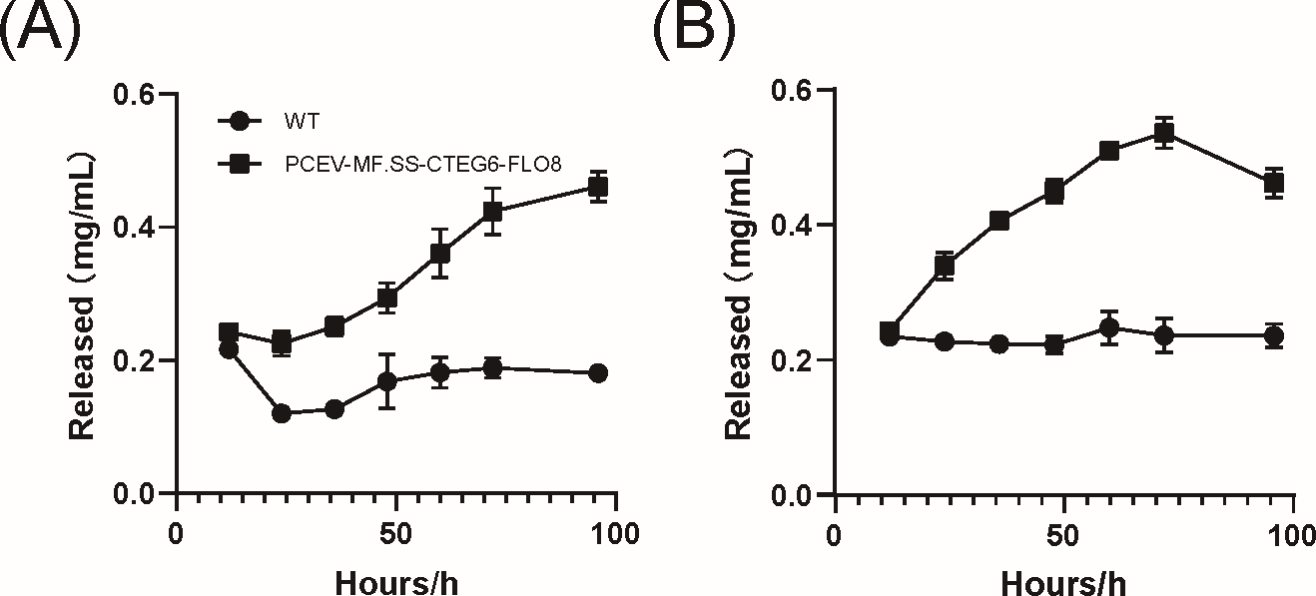
Fermentation of the engineered yeast strain displaying *CTEG6* at 30°C and pH 6 using different substrates. (**A**) Time course of reducing sugar released from 20 g/L MCC as the substrate during fermentation. (**B**) Time course of reducing sugar released from 20 g/L corncob powder during fermentation. Reducing sugars were measured by the DNS assay and are presented as mean ± SD (*n* = 3). Measurements are expressed in mg/mL.

To verify whether the engineered strain carrying *CTEG6* could also hydrolyze more structurally intricate cellulose, we conducted fermentations using microcrystalline cellulose (MCC) as a substrate. MCC, acid hydrolysis of natural cellulose, is primarily composed of linear polysaccharides linked by β-1,4-glucosidic bonds and exists as extremely fine, short rod-like or powdery porous particles. In plant fibers, MCC accounts for approximately 70% of the cellulose content and possesses greater structural stability and complexity than CMC-Na (49). During MCC fermentations, CTEG6 exhibited substantial hydrolytic activity: reducing-sugar titers increased steadily over time, significantly outpacing the wild-type control (Fig. 5A). Encouraged by these results, we next evaluated corncob powder, a natural cellulose source. The engineered strain released up to 0.53 mg/mL reducing sugar at 72 h and maintained stable, sustained activity throughout the fermentation (Fig. 5B). Together, these results demonstrate that *CTEG6*-expressing yeast can efficiently hydrolyze both highly crystalline MCC and more complex, natural cellulose under mild fermentation conditions, supporting its potential for further development and scale-up for biomass valorization.

### Structural determinants of CTEG6’s superior catalytic activity

**Fig 6:**
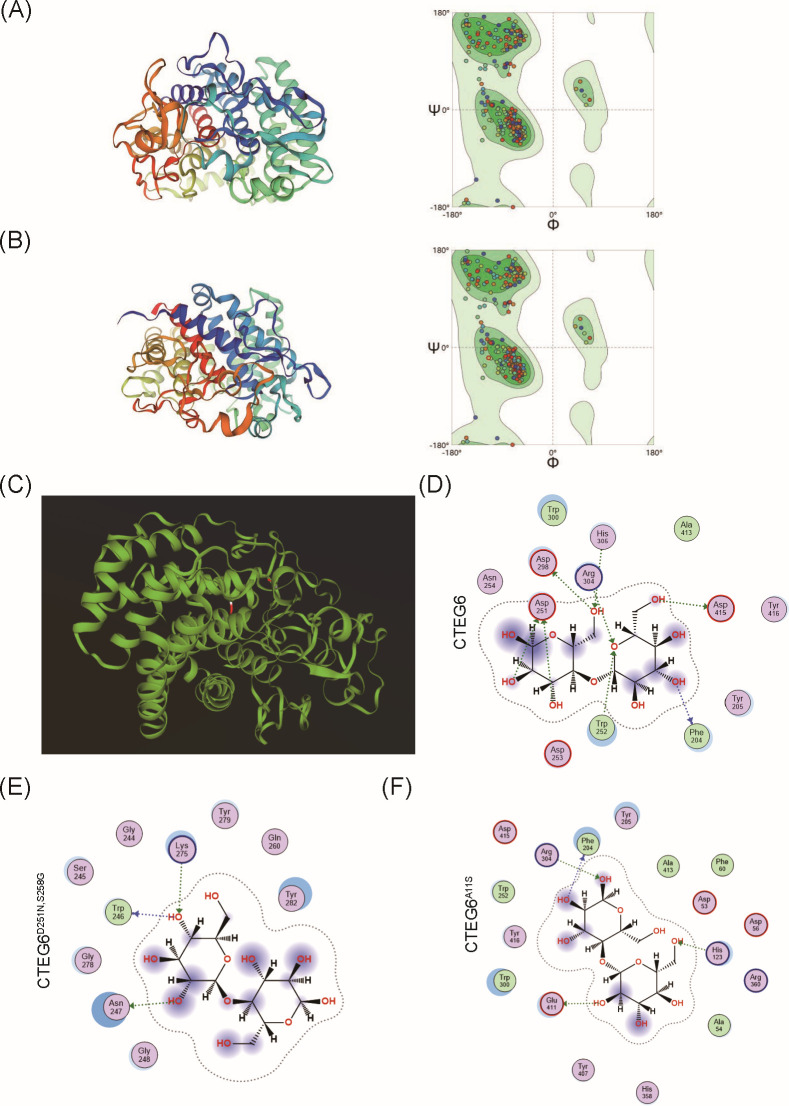
Molecular docking and structural analysis. Predicted three-dimensional structural diagram and corresponding Ramachandran plot for CTEG6 (**A**) and CTEG4 (**B**) (models generated with AlphaFold2; model quality assessed by Ramachandran analysis). (**C**) Structural overlay of CTEG6 and CTEG4, regions highlighted in red indicating structural differences in CTEG4. (**D–F**) Flexible, induced-fit docking of a representative CMC-Na fragment into wild-type CTEG6 and the double mutant CTEG6^D251N,S258G^ produced top poses with eight and two hydrogen bonds, respectively, and predicted binding free energies (ΔG) of −6.93 and −6.02 kcal/mol. (**F**) Modeling the A11S substitution reduced the hydrogen-bond count from eight to four and abolished the Asp251-mediated interactions, indicating that residue 11 influences the local geometry required for substrate engagement.

Among the eight EGs tested, CTEG6 displayed the highest hydrolytic activity, whereas CTEG4 was the least active. To investigate the structural basis for CTEG6’s superior performance, we modeled the three-dimensional folds of both enzymes using AlphaFold2 (MMseqs2 mode, v1.3.0; Fig. 6A and B). Sequence alignment identified only four residue differences between the two proteins (residues 11, 27, 251, and 258), and structural superposition showed that CTEG6 and CTEG4 adopt the same overall fold with only minor local deviations (Fig. 6C). Notably, CTEG3 and CTEG4 differ solely at residue 27, yet CTEG3 exhibited only approximately twofold higher activity despite having approximately a fourfold greater gene copy number, suggesting that Lys27 alone does not fully explain the activity gap. To probe alternative determinants, we carried out flexible, induced-fit docking of a representative CMC-Na fragment with wild-type CTEG6 and the double mutant CTEG6^D251N,S258G^ (50). Asp251 introduces a negatively charged carboxylate that forms direct hydrogen bonds with the glucan ligand, anchoring the substrate and improving electrostatic complementarity. Ser258 adds a side-chain hydroxyl that increases local polar contacts and reduces backbone flexibility, helping to stabilize the binding cleft. The top-ranked poses for wild-type CTEG6 formed eight hydrogen bonds with the ligand (two of which involve Asp251), whereas the double mutant formed only three hydrogen bonds; the predicted binding free energies (ΔG) were −6.93 and −6.02 kcal·mol^−1^, respectively (Fig. 6D and E). These data indicate that residues D251 and S258 contribute critically to substrate binding and stabilization. We additionally modeled the single A11S substitution in CTEG6, which reduced the hydrogen-bond count from eight to four and abolished the Asp251-mediated interactions, indicating position 11 influences the local geometry required for D251 to engage the substrate (Fig. 6F).

**Fig 7:**
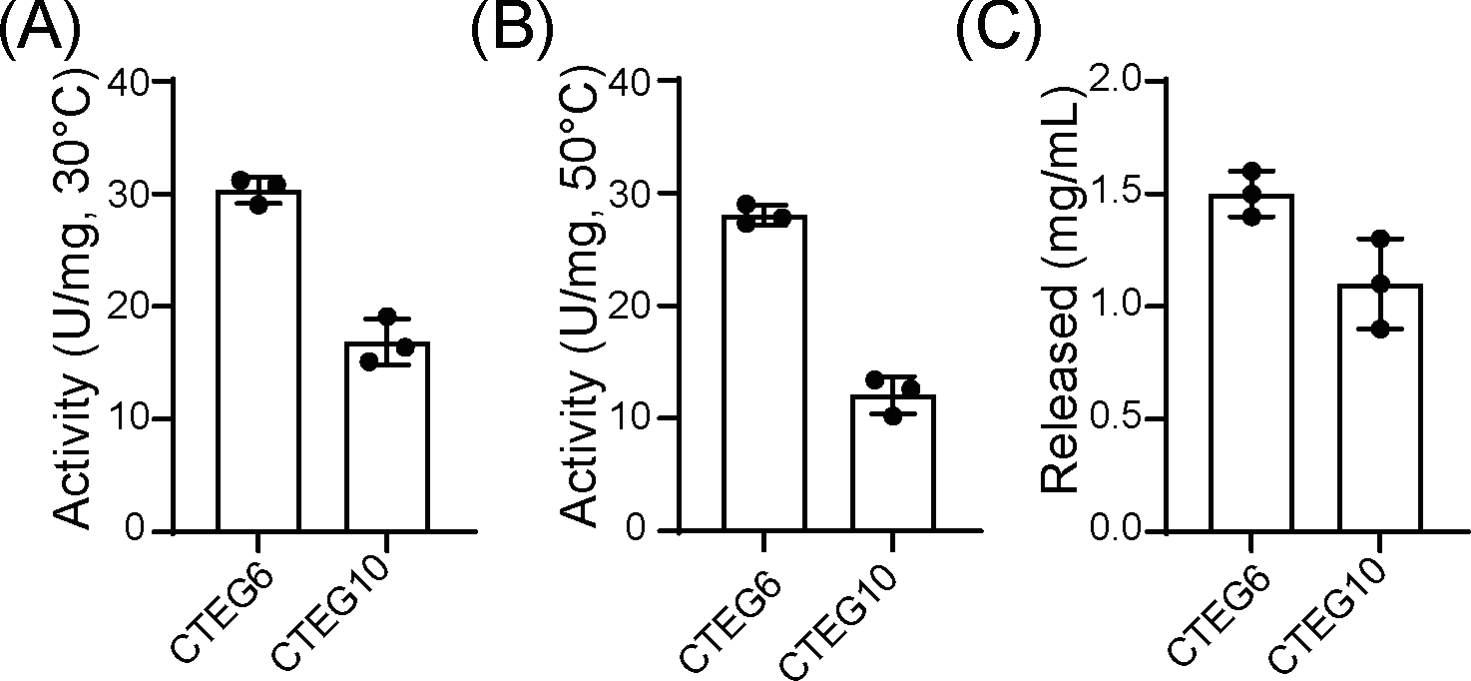
Experimental validation of docking-predicted roles of A11, D251, and S258 in CTEG6. Functional validation of CTEG6 mutations. (**A–B**) Specific activities of crude enzyme extracts from strains expressing wild-type CTEG6 and the triple mutant CTEG10 (CTEG6^A11S/D251N/S258G^) assayed on 20 g/L CMC-Na at 30°C (**A**) and 50°C (**B**). Activities were determined by the DNS assay and are reported as U/mg (per mg dry cell weight). (**C**) Reducing sugar titer after 72 h of shake-flask fermentation on 20 g/L CMC-Na for strains expressing CTEG6 and CTEG10. Data are presented as mean ± SD (*n* = 3).

To experimentally validate residues implicated by our docking and sequence analyses, we constructed the triple mutant *CTEG10* (*CTEG6^A11S,D251N,S258G^*) by nested PCR, confirmed its sequence by Sanger sequencing, and expressed both *CTEG6* (wild type) and *CTEG10* for side-by-side comparison. Crude extracts from three independent transformants of each construct showed reduced specific activities for CTEG10: 17.77 U/mg versus 30.33 U/mg for wild type at 30°C, and 12.27 U/mg versus 28.03 U/mg at 50°C (means of three biological replicates; Fig. 7A and B). Consistent with the *in vitro* assays, 72-h whole-cell fermentations on 20 g/L CMC-Na showed reduced hydrolytic performance for CTEG10: reducing-sugar titers declined from 1.50 mg/mL (CTEG6) to 1.10 mg/mL (CTEG10) (Fig. 7C). Together, the computational and experimental data implicate residues A11, D251, and S258 as cooperative determinants of substrate recognition and catalytic efficiency in CTEG6.

## DISCUSSION

Efficient depolymerization of cellulose into soluble oligosaccharides followed by their conversion into bio-based platform chemicals and polymers, would provide a viable strategy for harnessing renewable biomass resources in industry, as well as the food and livestock sectors (51). However, this goal has been hindered by the scarcity of efficient EGs and the absence of robust expression and fermentation systems. As a result, current cellulose degradation processes suffer from low conversion efficiency, severe pollution, and high operational costs, limiting their industrial adoption (52). Therefore, the discovery of potent EGs and the construction of effective expression cassettes are critical for improving the commercial viability of biomass bioconversion.

In this study, we identified eight novel EGs from a *Coptotermes formosanus* cDNA library and successfully anchored them on the surface of *S. cerevisiae* via the Flo8 flocculin anchor. This surface-display strategy markedly enhanced the cellulose hydrolysis capability of the engineered strains, enabling continuous and efficient degradation under mild conditions with enzyme reuse (53). The gene expression cassette we constructed enabled effective cellulose hydrolysis. Among the eight candidates, CTEG6 stood out for its superior catalytic activity, robustness, and stability, performing optimally at 30°C and pH 6. Notably, CTEG6 exhibited a broad substrate specificity and effectively hydrolyzed both artificial and natural celluloses, including CMC-Na, MCC, and corncob powder.

This study also explored the molecular basis of CTEG6’s strong hydrolytic performance through molecular docking simulations, offering insights for future optimization. Our structural analyses (AlphaFold2 models and flexible, induced-fit docking) pinpointed residues A11, D251, and S258 as molecular determinants of CTEG6’s superior activity. To validate these *in silico* predictions, we constructed a triple mutant (CTEG10: A11S, D251N, S258G) and evaluated its performance side-by-side with wild-type CTEG6. The mutant showed substantially reduced specific activities and lower whole-cell hydrolysis. These experimental results corroborate the docking predictions: wild-type CTEG6 forms a dense hydrogen-bonding network with a CMC-Na fragment, while the D251N/S258G substitutions markedly diminish hydrogen bonding and predicted substrate affinity. Modeling of the A11S substitution likewise reduced hydrogen bonding and disrupted D251 interactions, indicating that position 11 helps maintain the geometry necessary for D251 to engage the substrate. Together, the computational and mutational data support a model in which residues 11, 251, and 258 act cooperatively to stabilize substrate binding and promote catalysis.

Interestingly, the eight newly identified EGs exhibited varying levels of thermostability, suggesting that factors beyond catalytic activity may influence the fermentation performance of the engineered strains, such as thermal tolerance and expression stability. Selecting the appropriate CTEGn to specific reaction systems could therefore improve both efficiency and commercial viability. Previous studies have shown that fermentation outcomes can be affected by variables such as metal ion concentrations, buffering systems, and substrate pretreatment methods (54, 55). Accordingly, future efforts will focus on optimizing fermentation conditions and implementing substrate pretreatment strategies to further boost yields. Additionally, differences in catalytic domain architecture likely contribute to the functional diversity among EGs. A more in-depth and systematic characterization of these domains could yield valuable insights into structure-function relationships. Notably, the integration of cellulose-binding modules and specific adhesion proteins, such as oligosaccharide linker proteins, has been shown to significantly enhance enzyme-substrate interactions and overall hydrolytic efficiency (56). These strategies present promising opportunities for the rational engineering of next-generation cellulases with improved activity, substrate affinity, and industrial applicability.

Although our degenerate-primer strategy targeting conserved catalytic motifs effectively enriched the library for bona fide endo-acting EGs and kept downstream functional screening tractable, it inevitably biases discovery toward canonical architectures and may miss noncanonical enzymes with novel or superior properties. In addition, our current reliance on the DNS assay, while suitable for quantifying total reducing sugars, cannot resolve degree-of-polymerization or oligosaccharide composition and therefore does not fully define enzyme specificity. To address this limitation, we will perform time-course product profiling by HPLC (or ion chromatography) in follow-up studies.

To capture atypical yet potentially valuable catalysts, future work will combine primer-independent sampling (e.g., cDNA/metatranscriptomics or shotgun metagenomics) with high-throughput functional selection (yeast surface display or droplet microfluidics), followed by focused biochemical validation. In parallel, we will explore multienzyme self-assembly strategies, such as designer cellulosomes, to maximize catalytic synergy, offer a promising route to enhance saccharification through substrate targeting and proximity effects (57). While stoichiometry control, complex stability, and display efficiency remain practical challenges, integrating primer-independent discovery with on-cell, self-assembling multienzyme systems and high-throughput functional screening should accelerate identification of robust, application-ready cellulase consortia. Finally, to remove copy-number variability as a confounding factor in comparative studies, we plan to adopt targeted genomic integration or CRISPR-based single-copy insertion at defined loci to standardize expression and enable cleaner engineering.

In summary, our integrated discovery, structural, and engineering approach identified CTEG6 as a robust endoglucanase for yeast-based cellulose hydrolysis and pinpointed residues A11, D251, and S258 as key contributors to substrate binding and catalysis. These findings advance mechanistic understanding and offer concrete targets and strategies for rational enzyme engineering. By enabling more efficient conversion of agricultural and industrial residues into valuable oligosaccharides and platform chemicals, our work lays the groundwork for scalable, lower-impact bioprocesses with reduced environmental footprint of biomass utilization.

## Data Availability

The nucleotide sequences have been deposited in NCBI GenBank under accession numbers PX138761 and PX138763–PX138769 (*CTEG1* and *CTEG3*–*CTEG9*).
